# Targeting the Sphingosine Kinase/Sphingosine-1-Phosphate Signaling Axis in Drug Discovery for Cancer Therapy

**DOI:** 10.3390/cancers13081898

**Published:** 2021-04-15

**Authors:** Preeti Gupta, Aaliya Taiyab, Afzal Hussain, Mohamed F. Alajmi, Asimul Islam, Md. Imtaiyaz Hassan

**Affiliations:** 1 Centre for Interdisciplinary Research in Basic Sciences, Jamia Millia Islamia, Jamia Nagar, New Delhi 110025, India; fun.preets@gmail.com (P.G.); aaliyataiyab04@gmail.com (A.T.); aislam@jmi.ac.in (A.I.); 2 Department of Pharmacognosy, College of Pharmacy, King Saud University, Riyadh 11451, Saudi Arabia; afihussain@ksu.edu.sa (A.H.); malajmii@ksu.edu.sa (M.F.A.)

**Keywords:** sphingosine metabolism, sphingosine kinase, cancer therapy, kinase inhibitors, drug design and discovery

## Abstract

**Simple Summary:**

Cancer is the prime cause of death globally. The altered stimulation of signaling pathways controlled by human kinases has often been observed in various human malignancies. The over-expression of SphK1 (a lipid kinase) and its metabolite S1P have been observed in various types of cancer and metabolic disorders, making it a potential therapeutic target. Here, we discuss the sphingolipid metabolism along with the critical enzymes involved in the pathway. The review provides comprehensive details of SphK isoforms, including their functional role, activation, and involvement in various human malignancies. An overview of different SphK inhibitors at different phases of clinical trials and can potentially be utilized as cancer therapeutics has also been reviewed.

**Abstract:**

Sphingolipid metabolites have emerged as critical players in the regulation of various physiological processes. Ceramide and sphingosine induce cell growth arrest and apoptosis, whereas sphingosine-1-phosphate (S1P) promotes cell proliferation and survival. Here, we present an overview of sphingolipid metabolism and the compartmentalization of various sphingolipid metabolites. In addition, the sphingolipid rheostat, a fine metabolic balance between ceramide and S1P, is discussed. Sphingosine kinase (SphK) catalyzes the synthesis of S1P from sphingosine and modulates several cellular processes and is found to be essentially involved in various pathophysiological conditions. The regulation and biological functions of SphK isoforms are discussed. The functions of S1P, along with its receptors, are further highlighted. The up-regulation of SphK is observed in various cancer types and is also linked to radio- and chemoresistance and poor prognosis in cancer patients. Implications of the SphK/S1P signaling axis in human pathologies and its inhibition are discussed in detail. Overall, this review highlights current findings on the SphK/S1P signaling axis from multiple angles, including their functional role, mechanism of activation, involvement in various human malignancies, and inhibitor molecules that may be used in cancer therapy.

## 1. Introduction

Sphingolipid metabolites are important bioactive signaling molecules that play essential roles in cell physiology and are associated with various human diseases. Numerous functions, including cell growth, survival, apoptosis, lymphocyte trafficking and angiogenesis have been attributed to sphingolipid metabolites [1,2]. They play crucial roles in normal cell homeostasis and the initiation and progression of several diseases. Mainly, ceramide, sphingosine, and S1P form the central core of sphingolipid metabolism and control diverse physiological functions by regulating multiple signaling cascades [3,4]. 

Ceramide is a key metabolite of the sphingolipid metabolism whose synthesis occurs via various distinctive pathways that include the sphingomyelinase pathway, the *de novo* pathway, the salvage pathway, and the exogenous ceramide-recycling pathway [5]. Ceramide, catabolically synthesized from sphingomyelin, forms specific domains within the cell membrane’s lipid rafts called ceramide platforms. These unique membrane domains regulate critical cellular functions such as apoptotic signaling and viral or bacterial antigens’ entry into the cell [6]. The *de novo* synthesis of ceramide occurring in endoplasmic reticulum begins with the condensation of serine and palmitoyl-CoA via serine palmitoyltransferase, followed by the functional activity of 3-ketodihydrosphingosine reductase, dihydroceramide synthase, and dihydroceramide desaturase in a stepwise manner. The *de novo* synthesis of ceramide is metabolically induced by the increased levels of serine or palmitate or via heat stress and chemotherapeutic agents [5,7]. The elevated intracellular ceramide levels promote cell cycle arrest and consequent suppression of cell proliferation [8]. Ceramide also induces programmed cell death by activating caspases or stress kinases such as c-JUN N-terminal kinase (JNK) and p38 [9].

In contrast to ceramide, S1P acts oppositely by promoting cell proliferation and survival [10]. Within the cell, the levels of ceramide and S1P are in an intricate dynamic balance with each other and determine the cell’s fate. SphK catalyzes the synthesis of S1P from precursor molecule sphingosine is the key regulator of this balance called the sphingolipid rheostat [11]. The tipping of the sphingolipid rheostat in favor of S1P production causes uncontrolled cell proliferation leading to cancer and other inflammatory diseases, whereas an elevated ceramide level promotes cell cycle arrest. The up-regulation of SphK with subsequent accumulation of S1P within the cells is implicated in different disorders, including diabetes, Alzheimer’s disease, atherosclerosis, cancer and inflammatory disorders [12,13,14]. This makes SphK/S1P signaling axis a potential drug target to develop effective therapeutics to manage cancer and other diseases.

In this review, we discuss the recent progress in SphK and its association with various human pathologies highlighting their mechanism of action, activation, and regulation. The SphK/S1P/S1PR axis’s role in the pathophysiology of diseases focusing on different types of cancer is comprehensively reviewed. Finally, we discuss the SphK/S1P signaling axis’s current progress highlighting their underlying mechanism of action and regulation that could be useful in drug design and discovery studies.

## 2. Sphingolipid Metabolism

**Figure 1:**
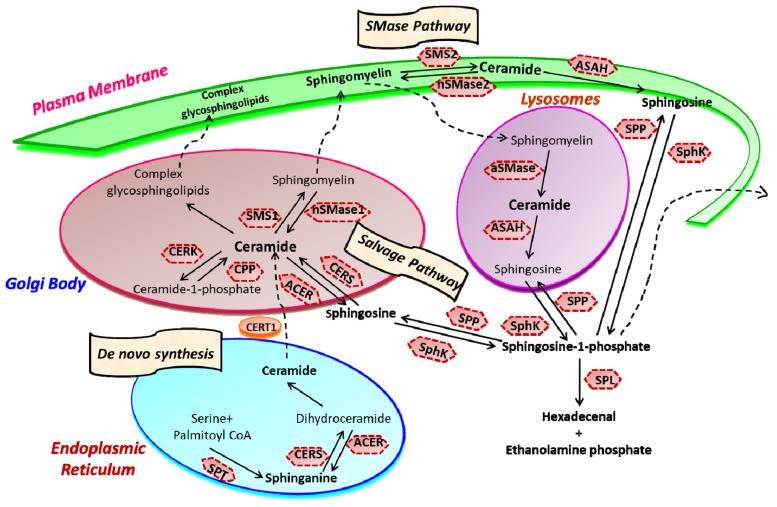
Sphingolipid metabolism and compartmentalization. Three significant mechanisms for ceramide generation are illustrated. Ceramide is synthesized via s “de novo pathway” in the ER from where it is transported to the Golgi bodies through CERT and serves as a substrate for the synthesis of complex glycosphingolipids (GLSL) and sphingomyelin (SM). GLSL and SM are transported to the plasma membrane through vesicular transport. In another mechanism; ceramide can be generated by the action of neutral or acidic SMases (“SMase pathway”) in the plasma membrane. Finally, in the “salvage pathway”, ceramide is synthesized from sphingosine released from the lysosome by the catalytic action of CERS. (SPT, Serine palmitoyltransferase; CERS, Ceramide synthase; CERK, Ceramide kinase; CPP, Ceramide-1-phosphate phosphatase; SMS, Sphingomyelin synthase; SMase, Sphingomyelinase; ACER, ceramidase in Golgi bodies; ASAH, ceramidase in lysosomes; SK, sphingosine kinase; SPP, Sphingosine-1-phosphate phosphatase; SPL, S1P lyase) (Figure is adapted from reference [18]).

Sphingolipid metabolism is a highly coordinated broad-spectrum cellular pathway, wherein several pathways are linked together. The synthesis and degradation of bioactive sphingolipids are regulated by several enzymes having fluxes of diverse metabolites [15]. Almost all the key enzymes of sphingolipid metabolic pathways have been identified which has unveiled the complexity of these pathways and their distinct subcellular compartmentalization [16,17]. An outline of the highly coordinated sphingolipid metabolism connecting several pathways is represented in Figure 1. The bioactive lipid ceramide occupies the central position in the sphingolipid metabolism hub and can be produced through diverse pathways via three primary mechanisms [1,2]. The first one is the de novo generation of ceramide that begins in the endoplasmic reticulum (ER) with 3-ketosphinganine from palmitoyl-CoA and serine units the catalytic action of serine palmitoyltransferase (SPT).

Ceramide synthase (CERS) catalyzes the acylation of dihydrosphingosine to form dihydroceramide which is converted to ceramide by desaturase [3,8]. Thereafter, ceramide can follow multiple intracellular routes. Ceramide is transported to the Golgi bodies by ceramide transport protein (CERT) where it is metabolically converted to sphingomyelin and various complex sphingolipids [2,19]. The transport of ceramide from ER to the site of sphingomyelin synthesis occurs via both vesicular and non-vesicular pathways [20,21].

In Golgi bodies, ceramide is converted to sphingomyelin, a vital component of the plasma membrane, by incorporating phosphocholine head group by sphingomyelin synthases (SMS). Glycosylation of ceramide by glycosyl or galactosyl CERS results in the formation of complex glycosphingolipids, an integral part of cell membranes and known to confer drug resistance to cancerous cells [22,23]. Additionally, ceramide can be directly phosphorylated to form ceramide-1-phosphate (C1P) by the catalytic activity of a specific ceramide kinase (CERK), which plays a crucial role in regulating cell homeostasis as well as in mediating inflammatory responses. C1P is further transported to the plasma membrane or other organelles for various biological signaling cascades by the C1P transfer protein. Conversely, ceramide can be generated through the catabolic degradation of sphingomyelin via sphingomyelinase (SMase), another mechanism of ceramide generation [24]. Three distinct forms of mammalian SMase having different pH optima i.e., acidic, neutral, and alkaline, has been identified [25]. While neutral SMase is responsible for generating ceramide in Golgi bodies (nSMase1) and plasma membrane (nSmase2). The acid SMase leads to ceramide synthesis from sphingomyelin in lysosomes (aSMase) [26,27]. In addition to that, the alkaline SMase is involved in the breakdown of dietary sphingomyelin in the intestine.

Finally, the third mechanism is the salvage pathway through which ceramide is generated from sphingosine by the action of CERS. Ceramide can be metabolized back to bioactive lipid sphingosine by specific ceramidase (CDase or ACER in Golgi bodies) and ASAH in lysosomes). Subsequently, sphingosine kinase (SphK or SK) catalyzes the phosphorylation of sphingosine to form another potent signaling molecule, sphingosine-1-phosphate (S1P). The S1P can either be degraded to ethanolamine phosphate and fatty aldehyde by lyase (SPL) or dephosphorylated to sphingosine by S1P phosphatase and re-acylated back to ceramide [1,2,4,5,24].

Ceramide and S1P are among the key sphingolipids having special biological functions. They function oppositely, with ceramide acting as a pro-apoptotic molecule controlling growth arrest and autophagy, whereas S1P functions as a pro-survival molecule and regulates cell proliferation, survival, angiogenesis, and cell trafficking. The dynamic balance of these two opposite-acting sphingolipids is referred to as the “sphingolipid-rheostat” regulating the cell’s fate [11,28,29]. Under normal physiological conditions, the balance of the sphingolipid rheostat is well maintained by the functional activity of a key enzyme, SphK. The bending of this intricate balance towards sphingosine induces cell cycle arrest and apoptosis. In contrast, the accumulation of S1P inside activates cell growth and proliferation pathways, often leading to pathological conditions such as cancer and other inflammatory diseases. The notion that the “sphingolipid-rheostat” defines the cell fate by controlling the levels of ceramide and S1P is further strengthened by the fact that inhibition of SphK leads to decreased S1P, and enhanced ceramide, ensuring cell death. 

One of the essential aspects of ceramide metabolism is the segregation of metabolic pools and bioactive metabolites within the cells, which seems crucial for ceramide to function as a regulatory signaling molecule. Such compartmentalization is facilitated by the distinct and well-defined subcellular localization of enzymes regulating ceramide metabolism [17,30,31]. Another key feature of ceramide metabolism is the translocation of ceramide from its synthesis i.e., the endoplasmic reticulum (ER) to the Golgi bodies, where it is transformed into sphingomyelin sphingosine and complex glycosphingolipids. Ceramide is converted to sphingosine by the catalytic activity of ceramidase (CDase) in the nucleus, plasma membrane, lysosomes, and mitochondria. Sphingosine is further converted to S1P by SphK1 in the cytoplasm and plasma membrane for intracellular and extracellular signaling, respectively [5,24,32]. The production of S1P in ER and mitochondria is facilitated by the catalytic action of SphK2, where it is localized [12,33].

## 3. Sphingosine Kinase

SphK is a lipid kinase that catalyzes the ATP-dependent phosphorylation of sphingosine to S1P [34,35]. Two isoforms of SphK—SphK1 and SphK2—have been characterized in humans that regulate various cellular processes [36,37]. They catalyze the same biochemical reaction but differ in their substrate affinities, tissue distribution, and subcellular localization [38]. SphK1 resides in the cytoplasm under normal physiological conditions, but it is translocated to the plasma membrane when activated. In contrast, SphK2 is present in the nucleus. Although the two isoenzymes are highly homologous, they have been observed to perform distinct functions. SphK1 shows pro-survival effects, whereas studies point towards a pro-apoptotic role of SphK2 [39,40,41].

### 3.1. SphK1 Activation and Functions

**Figure 2:**
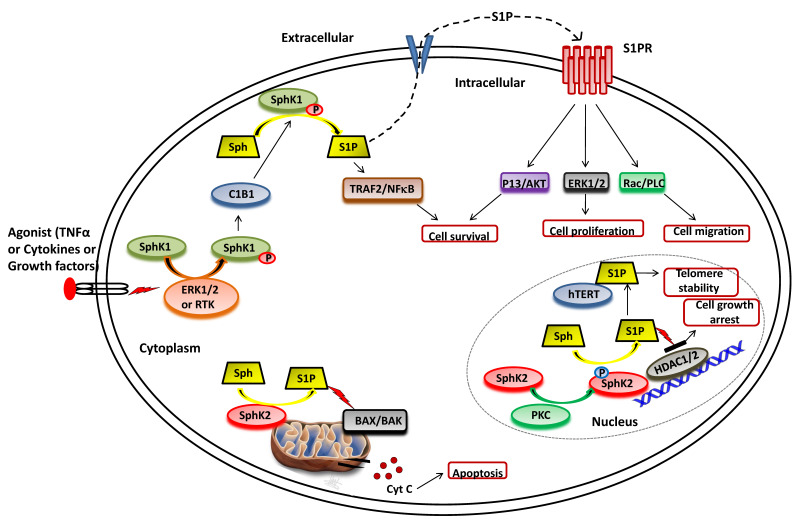
Functional roles of SphKs and S1P in cells. Upon ERK1/2 mediated phosphorylation/activation in the presence of various agonist (such as TNFα, cytokines and diverse growth factors), SphK1 is translocated to plasma membrane from cytoplasm and interact with calcium-myristoyl switch protein 1 (C1B1). This facilitates the phosphorylation of sphingosine to generate S1P, which can either be secreted out or interacts with intracellular targets (such as TRAF2) to elicit its functions. Once secreted out of the cell, S1P binds to the S1P receptor (S1PR) embedded in the plasma membrane and activates various downstream signaling pathways that control cell survival, proliferation, and migration. In the nucleus, SphK2 catalyzes the phosphorylation of sphingosine to generate S1P that inhibits the activity of histone deacetylases (HDAC1/2) and regulates gene expression. S1P also binds to human telomerase reverse transcriptase (hTERT) at the nuclear periphery in human and mouse fibroblasts that inhibits its interaction with makorin ring finger protein 1 (MKRN1) and promotes telomerase stability. S1P is also produced in the mitochondria by the action of SphK2.

The function of SphK1 and subsequent intracellular levels of S1P is regulated by various mechanisms, including gene transcription, translational regulation and posttranslational modifications [42,43,44]. In particular, the activity of SphK1 is regulated by various agonists and stimuli that facilitate its phosphorylation and translocation to the plasma membrane, where it synthesizes S1P from sphingosine (Figure 2). A range of external stimuli that induce phosphorylation of SphK1 includes transforming growth factor β (TGF-β), tumor necrosis factor α (TNF-α), pro-inflammatory cytokine, and various growth factors via the activation of receptor tyrosine kinases, G-protein coupled receptors and toll-like receptors [45,46,47]. TGF-β has been observed to induce SphK1 activity by upregulating its gene expression levels [48,49,50]. Moreover, TGF-β exposure reduces the activity of S1P phosphatase, ultimately leading to a transient and rapid increase in S1P intracellular levels [51,52]. Another activator of SphK1 is TNFα that enhances SphK1 activity in the human umbilical vein endothelial cell (HUVEC) [53,54]. Other endogenous agonists include prolactin and 17β-estradiol, both upregulate the expression and activity of SphK1 [55,56,57].

Numerous studies suggest that the molecular mechanisms of agonist-induced stimulation involve the direct phosphorylation of SphK1 at Ser225 by ERK1/2 followed by conformational changes in the lipid kinase. This phosphorylation is crucial for the increased SphK1 activity but is required to translate the enzyme from the cytoplasm to the plasma membrane [33,41,58]. Upon activation, SphK1 interacts with calcium-myristoyl switch protein 1 that further facilitates the phosphorylation of sphingosine to S1P on the plasma membrane [59,60,61]. 

Several lines of evidence have consolidated the notion that SphK1 promotes cell survival. The elevated intracellular SphK1 levels appear to play an essential role in uncontrolled cell proliferation and metastasis in various cancer cell types [11,49,57,62,63,64]. A correlation between the expressions of SphK1 with short patient survival has also been observed. Brocklyn et al. [65] have demonstrated that SphK1 expression is inversely correlated with patient survival in glioblastoma multiforme. Multiple studies have illustrated that the targeted inhibition of SphK1 activity can be considered a potential strategy to combat cancer [15,49,66,67,68,69]. Likewise, the down-regulation of SphK1 via targeted inhibition induces apoptosis and enhances the sensitivity of cancer cell lines towards chemo- and radiation therapy [70,71]. Following these reports, a study showed the enhanced apoptosis in cardiomyocytes that lacks SphK1 compared to wild-type control cells under hypoxic conditions. In this case, monoganglioside (GM-1) administration improves the survival of wild-type adult mouse cardiomyocytes subjected to hypoxic and glucose deprivation conditions; however, it showed no effect on SphK1-null myocytes indicates that the activation of SphK1 by GM-1 in wild-type cells leads to cell survival [72]. 

### 3.2. SphK2 Activation and Functions

As illustrated in Figure 2, SphK2 is primarily localized to the ER or associated with mitochondria [33,39]. It also shuttles in and out of the nucleus with the assistance of nuclear localization and nuclear exportation signals possessed by the enzyme [73,74]. Earlier studies suggested apoptosis-promoting effects of SphK2 in contrast to the pro-survival action of SphK1. The overexpression of SphK2 often enhances apoptosis and cell cycle arrest [39]. The pro-apoptotic effects of this isoform have been mediated via a putative BH3 motif that interacts with the pro-survival Bcl-2 family member, Bcl-xL [75,76]. Moreover, the mitochondrial localization of SphK2 with subsequent S1P synthesis has been shown to confer the BID-mediated activation of BAK that modulates the membrane potential with the consequential release cytochrome *c* [35,77,78]. 

It has been shown that S1P produced by SphK2 in the nucleus inhibits HDAC1/2 activity that leads to enhanced histone acetylation at a specific promoter (Figure 2). This amplifies the transcription of cyclin-dependent kinase inhibitor p21 and transcriptional regulator *c-fos* genes, which would likely contribute to cell growth arrest attributed to SphK2 [78,79,80]. In conjunction with these findings, Weigert et al. [81] have demonstrated that knockdown of SphK2 could impede the enhanced apoptotic rates in cancer cells induced by the administration of either TNF-α or staurosporine. The genetic ablation of SphK2 suppressed the tumor growth in breast tumor xenografts (MCF-7). Contrary to the notion that SphK2 has apoptotic functions, several experimental studies have suggested an essential role of this isoenzyme in cell proliferation and survival similar to SphK1 in cancer cells [82,83,84,85]. Xu et al. [82] demonstrated that SphK2 is up-regulated in human osteosarcoma tissues and promotes cellular growth. Silencing of SphK2 by targeted shRNAs induces cell apoptosis and inhibits osteosarcoma cell growth. In another study, Xun et al. [85] showed that targeting SphK2 with a small molecule inhibitor, ABC294640, induces cell growth inhibition and apoptosis colorectal cancer cells.

Other intracellular targets of SphK2 include TERT and prohibitin. Selvam et al. [86] have shown that S1P, produced by the activity of SphK2, binds to human telomerase reverse transcriptase (hTERT) at the nuclear periphery in humans and mice fibroblasts. The binding of S1P to hTERT inhibits its interaction with makorin ring finger protein 1 (MKRN1), an E3 ubiquitin ligase that tags hTERT for degradation. Thus, S1P binding to hTERT promotes telomerase stability, telomere maintenance, cell proliferation, and tumor growth. In the mitochondria, S1P is mainly produced by SphK2 and binds with high affinity and specificity to prohibitin 2 (PHB2), a highly conserved protein that regulates cytochrome-c oxidase assembly and mitochondrial respiration [87].

## 4. Sphingosine-1-Phosphate and Its Receptors in the Pathophysiology

**Figure 3:**
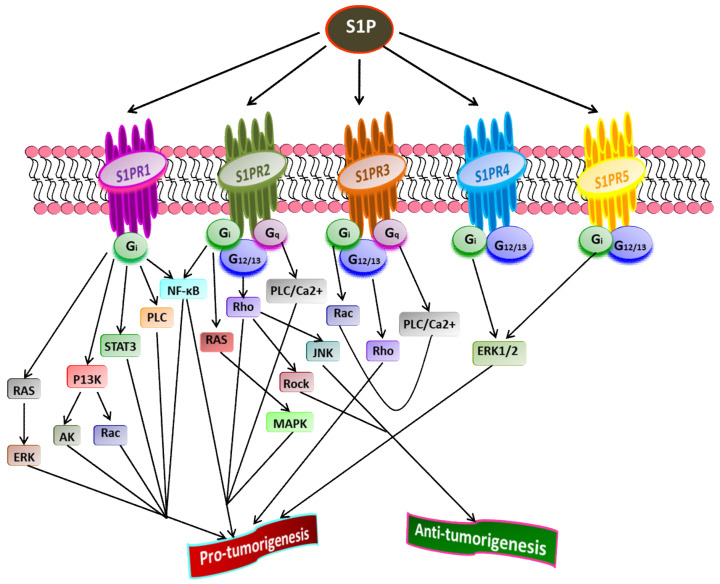
Role of S1P receptors in cancer. Once exported outside the cell, S1P binds and activates S1PR_1-5_ that further stimulates receptor-bound G-proteins (G_12/13_, G_q_, G_i_). Subsequently, the activated G-proteins turn on diverse downstream signaling pathways that play a crucial role in tumorigenesis. A cross-talk between various downstream targets of different S1PRs regulating cancer progression is evident (Adapted from [93]).

S1P acts as a second lipid messenger and regulates various physiological processes, crucial for both normal and pathological cellular conditions such as cell proliferation, migration, inflammation, and angiogenesis. S1P exerts its effects via binding to a family of five G-protein coupled receptors (GPCR), namely S1P receptor 1-5 (S1PR_1-5_) [15,43,88,89] (Figure 2). The autocrine or paracrine binding of S1P to S1P_1-5_ activates varieties of downstream signaling pathways that account for its role in many pathophysiological conditions, including cancer and inflammation [90,91,92]. Figure 3 illustrates different downstream targets of S1PRs that are vital for tumorigenesis. 

Liu et al. [94] have shown that binding S1P to S1PR1 in B cell lymphoma promotes tumor development. The inhibition of S1PR1 expression down-regulates STAT3 function leading to halting in tumor cell survival and invasion. High S1PR1 expression is associated with poor clinical outcome and the expression of several anti-apoptotic markers in non-muscle-invasive urothelial carcinoma [95]. In another study, Liu et al. [96] found the up-regulation of S1PR1 expression in bladder cancer tissues positively correlated with the density of tumor-infiltered specific regulatory T-cells in low survival rate in bladder cancer patients.

S1PR2 has been shown to promote cell proliferation and survival in acute myeloid leukemia and induce the expression of ezrin-radixin-moesin (ERM) proteins that encourage cell migration and invasion in cultured HeLa cells [97]. Recently, Kennedy et al. [98] found that S1PR2 promotes esophageal adenocarcinoma progression and cancer stem cell expansion via yes-associated protein (YAP) and β-catenin activation. Contrary to these findings, Petti et al. [99] have suggested that S1PR2 negatively correlates with the proliferation of epithelial cells in colorectal cancer. Complementing this study, Stelling et al. [100] showed the tumor-suppressive function of S1PR2 through TGFβ/TGF-β2/SMAD1 signaling axis in diffuse large B-cell lymphoma.

S1PR3 is overexpressed in breast cancer cell lines, promoting cancer progression and reducing the overall survival rate in breast cancer patients [62,101]. Hirata et al. [102] established the role of S1P/S1PR3 signaling with subsequent Notch activation in promoting tumorigenicity in aldehyde dehydrogenase-positive cancer stem cells. Moreover, they showed that tumor development in cancer stem cells could be inhibited by administering S1PR3 antagonist or S1PR3 knockdown. Recently, Shen et al. [103] found that S1PR3 is upregulated in osteosarcoma and that S1P/S1PR3 axis promotes cell proliferation and aerobic glycolysis in osteosarcoma cells. Furthermore, they showed a synergistic inhibitory action of methotrexate and S1PR3 antagonist TY52156 on osteosarcoma cellular growth. 

Less is known regarding the functions of S1PR4 and S1PR5 in pathophysiology compared to other S1PRs. However, the current findings suggest that both S1PR4 and S1PR5 are coupled to G_i_ and G_12/13_ [104,105,106]. S1P/S1PR4 signaling activates downstream targets, ERK1/2, and human epidermal growth factor receptor 2 (EGFR2) pathways, thereby increasing tumorigenicity. A positive correlation was found between the overexpression of SIPR4 and shorter survival in estrogen receptor (ER)-negative breast cancer patients [101,107]. A recent study revealed the enhanced mitotic progression in HeLa cells and chromosome segregation effects through S1PR5-dependent activation of the PI3K/AKT pathway [108]. S1PR5 has been implicated in potentiating cell survival in prostate cancer cells by inducing autophagy under serum-deprived conditions [109]. In contrast, S1PR5 expression is down-regulated in esophageal squamous cell carcinoma than in normal esophageal epithelium [110]. Additionally, S1PR4 and S1PR5 play a crucial role in inflammation, often linked to the progression of some cancers, such as colon cancer [111]. Overall, the downstream signaling pathways activated by S1P through S1PR_1-5_play central roles in regulating the cell proliferation, survival, migration, and invasion of cancer cells [93].

Although several studies have focused on S1P signaling through S1PRs; various lines of evidence suggest the presence of direct intracellular targets of S1P. Histone deacetylases, HDAC1 and HDAC2, were among the first identified intracellular targets whose activity is inhibited by the binding of S1P, and accounts for the epigenetic regulation of specific genes inside the nucleus (Figure 3) [14,79]. Subsequent studies illustrated the binding of other intracellular proteins to the S1P, such as TNF receptor-associated factor 2 (TRAF2); an E3 ubiquitin ligase that is a key mediator the NF-κB pathway induced by inflammatory signaling molecule TNF-α [112,113]. S1P has also been shown to modulate the activity of β-amyloid precursor protein cleaving enzyme 1 implicated in Alzheimer’s disease [114]. It is now well established that S1P mediates its functions via both extracellular and intracellular signaling pathways. However, more research has to be undertaken to unravel its intracellular targets.

## 5. SphK1/S1P Signaling in Human Malignancies

Evidence linking SphK/S1P signaling axis to cancer and other inflammatory diseases is comprehensive and conclusive. The up-regulation of SphK1 is seen in many human cancers, pointing out the possibility that its overexpression accompanied a rise in S1P synthesis that accounts for the pro-inflammation in the tumor microenvironment. SphK and S1P promote cancer progression in diverse ways by contributing to cell survival, proliferation, angiogenesis, apoptosis, and metastasis. SphK1 is often correlated with high clinical-grade tumors, resistance to radio- and chemotherapy, and poor prognosis and survival rate in patients. In the next part of this review, we focus on the current knowledge of SphK1/S1P signaling in various cancer types and other human pathologies. Further, we discuss the recent advancements in potential therapeutics targeting the SphK/S1P/S1PR signaling pathway.

### 5.1. Breast Cancer

**Figure 4:**
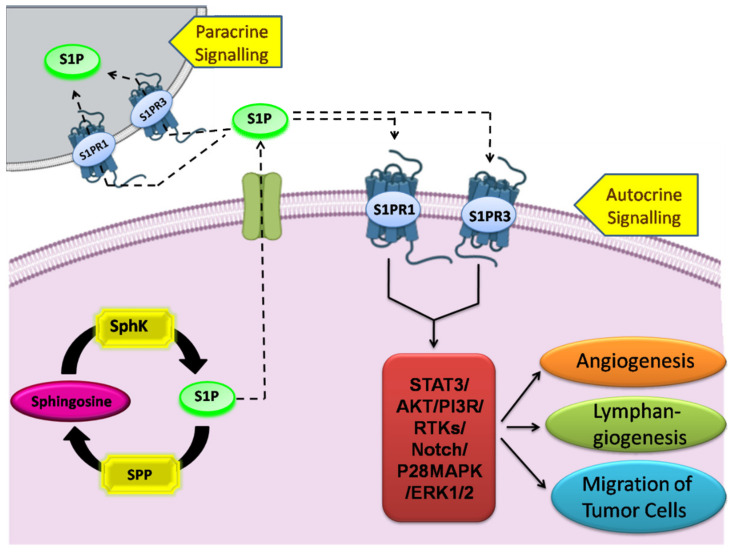
Schematic representation showing the role of the SphK1/S1P axis in breast cancer progression and metastasis. S1PR1 and S1PR3 are the two S1P receptors playing a major role in breast cancer development which initiate a cascade via activating STAT3, various RTKs, Notch, p38MAPK, ERK1/2, and AKT/PI3K cumulatively resulting in breast cancer metastasis.

Activation of SphK1 subsequently forms S1P, which binds to the S1P receptors (S1PR1 and S1PR3) in the autocrine/paracrine process of breast cancer signaling. As shown in Figure 4, the binding of S1P to S1PR1 resulted in persistent signal transducer and activator of transcription 3 (STAT3) activation promoting cancer cell progression [115]. The adipokine leptin, a product of adipocytes, upregulates STAT3 and SphK1 in ER-negative breast cancer cells. The activity and expression ofSphK1 via leptin are regulated by ERK1/2 and *Src* family kinase pathways [115]. Epidermal growth factor receptor, vascular endothelial growth factor receptor, and platelet-derived growth factor receptor are some of the receptor tyrosine kinases (RTKs) that get activated when S1P binds to S1PR1, which makes them significant in the process of angiogenesis and proliferation of breast cancer cells. Vascular endothelial growth factor-mediated activation of SphK1 is linked with the regulation of angiogenesis and lymph angiogenesis. Similarly, epidermal growth factor activation of SphK1 is significant for the migration of breast cancer cells [115]. 

Intriguingly, S1PR3 activation promotes Notch signaling and p38MAPK pathways resulting in proliferation and tumorigenicity, as shown in Figure 4 [102]. Most of the S1PR3’s breast cancer effects are due to the constant activation of ERK1/2 and AKT/PI3K pathways [93]. The inhibition of SphK1 leads to the suppression ofERK1/2 and AKT/PI3K phosphorylation in triple-negative MDA-MB-231 cell line resulting in decreased cell proliferation and survival meddling PKC activity and cytokinesis [116]. 

### 5.2. Lung Cancer

The mortalities due to lung cancer are highest among all cancer deaths [117]. SphK1 is significantly expressed in the non-small cell lung cancer (NSCLC) cells corresponding to tumor growth and the poor survival rates of NSCLC patients. Overexpression of SphK1 inhibits apoptosis while inducing antiapoptotic proteins c-IAP2, c-IAP1, TRAF1, and Bcl-xl in cancer cells. Up-regulation of SphK1 activates the PI3K/AKT/NF-κB pathway enhancing invasion and migration while inhibition of SphK1 suppresses the metastasis in NSCLC cells [118]. The overexpression of SIPR3 increased the EGFR expression in lung adenocarcinoma cell lines and SIPR3 knockout abrogates S1P-dependent EGFR activation. The ectopic expression ofS1P transporter Spns2 in NSCLC cells facilitate apoptosis via modulating GSK-3β and STAT3 pathways [119]. Gachechiladze et al. [120] showed that the immunohistochemical detection of SphK1 serves as a predictive biomarker in NSCLC patients’ patient’s platinum-based chemotherapy treatment. SphK1 expression is found to be elevated in malignant pleural mesothelioma, a resistant form of cancer that develops in the lining of the lungs. Sphingosine inhibits the mesothelioma cell lines growth via inhibiting PKC-δ, leading to the induction of cell cycle arrest at the G0/G1 [119]. 

SphK1/S1P signaling activates Hippo/YAP pathway and mtROS, and plays a significant role in pulmonary fibrosis, in which lungs become scarred over time and can lead to lung cancer. Hippo pathway-YAP regulates endothelial cell proliferation, migration and survival, concomitantly regulating vascular sprouting, vascular barrier formation, and vascular remodeling. Major intracellular signaling programs that regulate angiogenesis simultaneously activate YAP to regulate key events in angiogenesis. TGF-β induces activation of SphK1 in human lung fibroblasts which subsequently increases the YAP1 expression and its translocation to the nucleus. Moreover, activated SphK1 mediates the enhanced mtROS production and expression of fibronectin and α-smooth muscle actin, leading to fibroblast activation, which has a driving role in pulmonary fibrosis [121].

### 5.3. Colorectal Cancer 

Colorectal cancer is a significant health burden globally [122,123]. SphK1 catalyzes the formation of S1P followed by its transport from the cells by different transporters. SPNS2, a crucial transporter involved in colorectal cancer, enhances the release of intracellular S1P and increases S1PR3 expression, followed by activation of AKT and ERK pathways leading to proliferation, migration, invasion and apoptotic resistance in CRC cells [124]. The inhibition of HIF-1 via SphK1, targeting the hypoxia pathway, enhances the outcome of chemotherapy with 5-fu in patients who have colorectal cancer at the initial phase [125].

**Figure 5:**
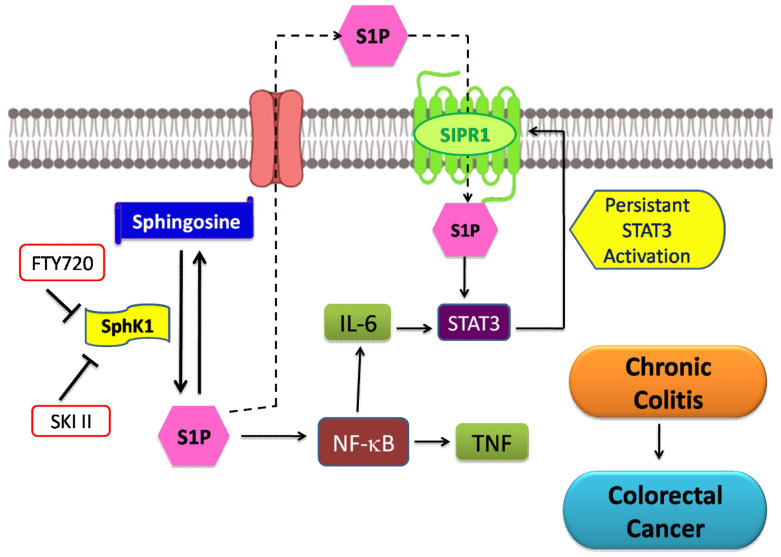
SphK1 and S1P signaling in colorectal carcinogenesis and progression. S1P produced by SphK1 results in the activation of NF-κB and IL-6/STAT3/AKT pathway intracellularly that prevents apoptosis, ultimately leading to cell transformation and angiogenesis in colitis-associated cancer and chronic intestinal inflammation, whereas signaling from S1PR1 maintains persistently activated STAT3 and up regulation of SphK, which contributes to the overall increase in S1P pool. Also, SphK inhibitors, SKI-II and FTY720 are known to suppress the progression of colon cancer.

Another study shows that sphingosine, which is phosphorylated by SphK1, resulting in the formation of S1P moiety, via regulating β-catenin levels inhibits the proliferation of cancer cells and induces cell death in SW480 and T84 colon cancer cell lines. It downregulates the expression of cyclin-dependent kinase 4 and suppresses phosphorylation of retinoblastoma protein, both being the principal cell cycle regulators [126]. As shown in Figure 5, S1P induces inflammatory signaling via activating NF-κB and IL-6/STAT3 pathways intracellularly, which prevents apoptosis and enhances cell proliferation and angiogenesis colitis-associated cancer and chronic intestinal inflammation. Also, STAT3-mediated pathways were found to promote S1PR1 signaling through positive autocrine-loop signaling, where persistent activation of STAT3 was associated with significantly elevated levels of S1PR1 in epithelial cells in the inflamed colon. Multiple studies have demonstrated that the cross talk of STAT3 and SphK-S1P-S1PR pathways may play an essential role in inflammation-induced tumorigenesis and tumor progression in the intestine [127]. SPL irreversibly degrades S1P, is downregulated in colon cancer. SGPL1 has been found to regulate a STAT3-activated cell transformation mediated by miRNA during colon carcinogenesis [126].

The SphK1/S1P pathway has been linked to the arachidonic acid cascade, particularly COX-2 and prostaglandin E_2_(PGE_2_), causing inflammation-related colon tumorigenesis. Down-regulation of SphK1 reduces the expression of COX-2 and the production of PGE_2_ levels in HT-29 cells belonging to human colon cancer. The evidence suggests that COX-2 knockdown reduces colon tumorigenesis whereas PGE_2_promotes the process. SphK1 deficiency resulted in reduced proliferation of cells and intensified apoptosis in AOM/DSS-induced colon cancer [128].

### 5.4. Gastric Cancer

In gastric cancer, S1P, SphK1 synthesizes binds to S1PR2 and transactivates two potent RTKs, EGFR and c-Met. The dysregulation of RTKs by overexpression of receptors and ligands has been a causative factor in gastric cancer progression. EGFR is not just responsible for transducing EGF signals, but it integrates various stimuli such as LPA, bombesin, thrombin, and S1P [129]. LPA-induced EGFR transactivation contributes to the activation of ERK1 which in turn phosphorylates C/EBPb, leading to cell proliferation and motility in GC cells and tissues. c-Met overexpression results in proliferation, enhanced survival, modified motility, and increased invasion of the cells into the extracellular matrix [129,130].

SphK1 overexpression suppresses the expression level of FoxO3a, thereby decreases Bim, a pro-apoptotic protein expression, resulting in enhanced apoptotic resistance in gastric cancer cells. SphK1 repression diminishes the ability to resist UV-induced cell death, implying SphK1 contribution in sustaining the unwanted survival of gastric cancer cells under radiotherapy. Down-regulation of FoxO3a by SphK1 is associated with AKT phosphorylation, suggesting a novel signaling cascade linking SphK1 to the antiapoptotic property of GC cells [131]. The down-regulation of SphK1 by locked nucleic acid–antisense oligonucleotides (LNA–ASO) resulted in considerable inhibition of GC cell growth, activated apoptosis, and cells were shown to be sensitized to doxorubicin [132]. miR-330-3p restricts tumor growth in gastric cancer mice model by directly targeting SphK1andS1PR1and suppressing their expressions and downstream ERK/AKT pathway. The expression levels of SphK1 were significantly escalated in GC tissues and cells, together with an elevated increase in SphK1-generated S1P concentrations in both GC serum and tissue [133]. Gastric cancer peritoneal dissemination is the prevalent form of metastasis in gastric cancer. Overexpression of SphK1 in human peritoneal mesothelial cells is linked to LC3B expression (an autophagy protein marker) which regulates human peritoneal mesothelial cells autophagy via TGF-β1 secretion in gastric cancer peritoneal dissemination progression. The in vivo study to establish the interactions between HPMCs and GC cells was carried out in a BALB/c nude mouse with SGC-7901 cells injected intraperitoneally and mixed with shSphK1 or shCtrl HPMCs. The results demonstrated that SGC-7901 cells co-injected with shSphK1 HPMCs exhibited reduced macroscopic nodules during peritoneal cavity dissemination to the mesenterium, greater omentum, and parietal peritoneum. In addition, the SGC-7901/shSphK1-HPMC tumor weight was significantly lower than that of the matched tumors in which SphK1 expression was not suppressed [134].

### 5.5. Glioma

A high level of S1P in the brain enhances glioblastoma cell adhesion and promotes mobility and invasiveness via S1P1-S1P3 receptors. SphK1 inhibition by SK1-I suppresses LN229 and U373 glioblastoma cell lines by initiating apoptosis and reducing tumor vascularization. SK1-I inhibits S1P, increases the levels of proapoptotic precursor ceramide, and targets p70S6K and GSK3β via suppressing the AKT pathway, which is often active gliomagenesis [135]. It has been reported that S1P induces the expression of urokinase plasminogen activator (uPA) and CCN1 (Cyr61) protein which together results in glioma invasion, growth, and angiogenesis. In contrast, the inhibition of SphK1 actively blocked the uPA system and thus repressing glioma invasion [136].

The high mRNA level of SphK1 is linked with high IL-1 expression in the U373 glioblastoma cell line and primary human astrocytes. IL-1 and NF-κB signaling are constitutively active in numerous cancers, including gliomas [137]. Glioma-secreted S1P maintains the anti-inflammatory phenotype of microglia to promote tumor progression and invasion [138,139]. Some studies have reported the increased SIPR2 levels during cerebral ischemia-reperfusion injury, implying the potential of S1P/SphK axis as a drug target in the treatment of cerebral ischemia-reperfusion injury [138,139].

## 6. Targeting SphK1/S1P Signaling Axis for Cancer Therapy

**Figure 6:**
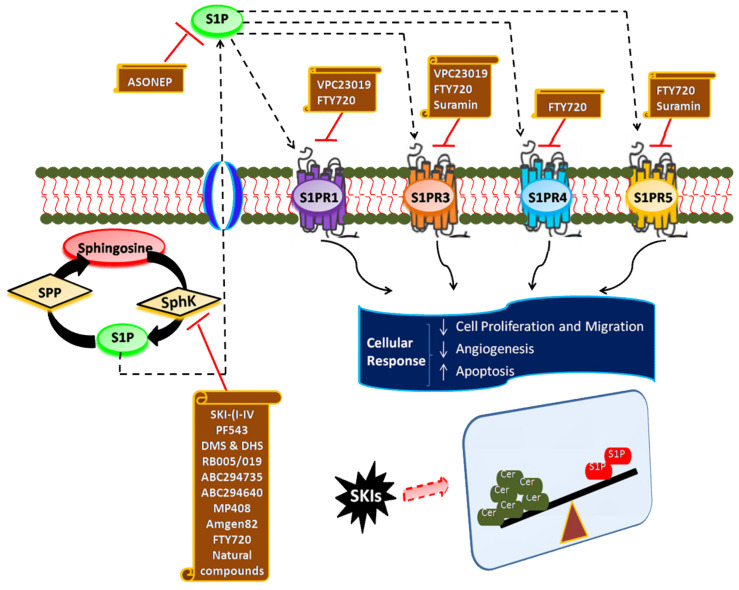
Compounds targeting SphK/S1P/S1PR signaling cascade and mediating anticancer activity. The up-regulation of SphK is observed in various tumor types and is linked to cancer progression. Enhanced SphK activity leads to elevated intracellular levels of S1P and subsequent over-activation and functioning of S1PRs, finally resulting in a marked increase in cell proliferation, angiogenesis, and metastasis. Several compounds (SKIs) that target and inhibit SphK/S1P/S1PR signaling axis are shown. They cause the tipping of sphingolipid rheostat towards the pro-apoptotic ceramide side leading to inhibition of cancer progression. Thus, SKIs can act as potential cancer therapeutics after proper evaluation and validation in efficacy and safety.

Given a well-established role of SphK/S1P signaling in cancer and other inflammatory diseases, small molecule inhibitors of SphKs, antagonists of S1P receptors, and S1P-blocking antibodies can serve as promising candidates in cancer therapeutics. Despite a high level of interest in this area of research globally, few inhibitory molecules have been identified to date. This area particularly suffers from the lack of selectivity of inhibitors towards SphK, no SphK inhibitor is in clinical use for the treatment of cancer. Any SphK1 inhibitor should encompass specific important characteristics for it to be used as an ideal drug molecule. These include high specificity and selectivity towards SphK1, effectiveness at low concentration (nM range), metabolic stability with no toxicity or minimal side effects. To date, no such inhibitor that fulfills all these requirements has been recognized. This section discusses the potential inhibitors of the SphK1/S1P signaling axis in different stages of laboratory research, biological testing (in vitro studies), animal model studies (in vivo studies) clinical trials. Figure 6 and Table 1 provide a detailed overview of these compounds targeting SphK/S1P/S1PR signaling cascade and mediating anticancer activity.

## 7. Inhibitors of SphK

### 7.1. SKI-(I-IV)

SKI-(I-IV) are first identified as non-lipid and non-selective small molecule SphK inhibitors [196]. Among all, SKI-II (4-((4-(4-chlorophenyl)–1,3-thiazolyl)amino)phenol) is a well-characterized and studied compound that acts as a competitive inhibitor binding to the lipid-binding pocket of SphK without affecting ATP-binding [34]. It shows good oral bioavailability and can reside in the blood for at least 8 h [196]. SKI-II is known to inhibit tumorigenesis in xenografts and mice models (IC_50_ = 0.5 µM) and induces apoptosis with antiproliferative effects in different cancer cell lines (IC_50_ = 0.9–4.6 µM) [71,130,197]. SKI-II exerts its effects through cathepsin B-associated lysosomal degradation of SphK1. However, speculations regarding its additional targets are also reported [198]. Recently, Grbčić et al. [142] reported the synergistic effect of co-administration of 5-FU and SKI-II in inhibiting cell proliferation, cell migration, and the clonogenic survival in HepG2 cells. Administration of SKI-II has been shown to inhibit fibrogenesis and improves liver injury in BDL or CCl4-treated mice model of hepatic fibrosis. It reduces the levels of transaminases and the downregulation of various fibrotic markers, including collagen I and α-SMA [143]. 

SKI-I is a selective inhibitor of SphK1 and is more potent than SKI-II, and inhibits ERK2 [196,199]. Another SphK1-specific analog of SKI-I i.e., SKI-178, with improved pharmacological properties has been developed (IC_50_ = 0.1–1.8 μM) which induces cytotoxicity in the acute myeloid leukemia cell lines through ceramide-induced and mitochondria-dependent apoptotic cell death [140,141]. The therapeutic potential of both SKI-II and SKI-I is compromised due to their restricted bioavailability and poor aqueous solubility. To overcome these issues, aspirinyl derivatives of both compounds have been designed to show similar or lower IC_50_ values compared to their chemical prototype [199].

### 7.2. PF543

PF543 is a novel selective inhibitor of SphK1 (IC_50_ of 3.6 nM). PF543 is a competitive inhibitor of sphingosine and shows100-fold higher selectivity for SphK1 over the SphK2 and other lipid and protein kinases and S1P receptors [200,201]. PF543 down-regulates the endogenous levels of SphK1 and S1Pand is helpful in identifying the specific roles of SphK1-driven S1P signaling. Administration of PF543 inhibits SphK1/S1P signaling and fibrogenesis in hepatic fibrosis mice model and human hepatic stellate cells (HSCs) by inducing impairment of collagen and α-SMA expression in TGF-β1-activated LX-2 cells [144]. It inhibits cell proliferation and imparts cytotoxicity to a series of colorectal carcinoma cell lines (HT-29, HCT-116, and DLD-1) [145]. Hamada et al. [146] showed that the treatment of PF543 induces cell death by promoting apoptosis, necrosis, and autophagy in SphK1-expressing head and neck squamous cell carcinoma (SCC) cells in a dose-dependent manner. Recently, Sun et al. [147] demonstrated that PF543 administration improves the clinical symptoms of ulcerative colitis in a mice model by reducing the S1P levels and inhibiting the expression of proinflammatory factors (IL-6 and IL-1β). Despite the wealth of literature on the anticancer properties of PF543, some studies show a contrary effect of PF543. In a study, Schnute et al. [201] showed specific inhibition of SphK1 with dramatically decreased levels of S1P but did not affect the proliferation and survival of 1483 head and neck carcinoma cells. 

### 7.3. N,N-Dimethylsphingosine, and Dihydroxysphingosine

DMS and DHS are sphingosine analogs and competitive inhibitors of SphKs having IC_50_ values of around 5 and 10 µM, respectively [202,203,204]. Both compounds show growth inhibition and induce apoptosis in human cancer cell lines [148,149,150,205]. These sphingosine analogs curb the radiation and chemo-resistance in several solid tumor cell lines [206,207,208]. DMS shows a similar inhibitory effect on both the isoforms of SphK non-selectively [150,209]. Chen et al. [150] demonstrated that DMS treatment triggers the apoptosis of human lung cancer cells (A549) by suppressing SphK1 activation. Zheng et al. [149] suggested that DMS blocks the TNF-α mediated expression of SphK1, hence E-cad in breast cancer cell line (MCF7) hindering cell growth and invasion. Another study by Zhang et al. [151] showed that DMS administration inhibits cell proliferation, invasion, and migration in hepatocellular carcinoma cells (SK-Hep1 and MHCCLM3) by suppressing the phosphorylation of AKT and NF-κB in these cells. 

DHS (safingol) acts as a substrate for SphK2 and competitively inhibits SphK1 [210,211]. Interestingly, Gude et al. [152] demonstrated that both DMS and DHS induce apoptosis in Jurkat and leukemia cells (U937), but rather than merely inhibiting SphK1 and SphK2, they dramatically up-regulate the expression of SphK1. This up-regulation has resulted in increased S1P levels that act as potent chemo-attractant of monocytes and macrophages. Both DMS and the DHS suffer from poor selectivity having multiple off-target, including other protein kinases, and often show severe toxic effects [212]. In combination with cisplatin and doxorubicin, DHS had reached a phase I clinical trial where it showed no indication of toxicity (NCT00084812) [153].

### 7.4. FTY720

FTY720 (fingolimod) is a structural analog of sphingosine, which was designed based on the structure of myriocin, a metabolite of fungus *Isariasinclairii* (myriocin) [213]. FTY720 is an FDA-approved potent immunosuppressant currently used to treat multiple sclerosis. Inside the cell, FTY720 elicits immunosuppressive effects once it gets phosphorylated by SphKs causing the internalization of S1P receptors, subsequently leading to lymphopenia [214,215,216]. The dephosphorylated form of FTY720 has antiproliferative and antimetastatic effects [217,218]. The antiproliferative potential of FTY720 is reported in diverse cancer cell types, including liver cancer, prostate cancer, breast cancer, ovarian cancer, bladder cancer, glioblastoma, leukemia, and malignant mesothelioma [154,155,156,157,158,159,160,219]. FTY720 shows anticancer properties via diverse mechanisms; a widely studied one involves the inhibition of SphK1, whose up-regulation leads to cancer progression, migration, and metastasis [188,220].

Another possible mechanism for anticancer activity of FTY720 includes inhibition of P13K/AKT/mTOR signaling and pro-survival MAPK pathway and upregulation of stress-activated kinases such as p38 that mediates the development of various types of cancer [218]. There is evidence that FTY720 functions through S1P receptor-independent mechanisms such as induction of ROS generation with subsequent apoptosis in various cancer cell lines. FTY720 inhibits the anti-apoptotic proteins, including Bcl-xl, Bcl-2, and Mcl-1, while increasing the pro-apoptotic protein Bax in the cell. This leads to the changes in mitochondrial membrane permeability with the subsequent release of cytochrome *c* [218,221,222,223]. FTY720 is reported to cause undesirable effects, possibly due to its interaction with more than one S1P receptor. The anticancer properties of FTY720 are primarily independent of effects on S1P receptors; one strategy to reduce the adverse side effects is to develop analogs, such as OSU-2S. They function without interacting with S1P receptors and hence do not induce immunosuppressive effects, which are mainly responsible for the risk of adverse events on the administration of FTY720 [160,188,224].

### 7.5. RB-005 and RB-019

Based on the FTY720scaffold, a series of SphKs inhibitors were synthesized. Among all the series, RB-005 and RB-019 show very high selectivity for SphK1 over SphK2. RB-005, being the most effective inhibitor with an IC_50_ = 3.6 µM, induces the proteasomal degradation of SphK1 [161,209,225]. The difference in the location of the hydroxyl group on piperidine moiety is believed to be the probable reason for less potency of RB-019 over RB-005 [225].

### 7.6. ABC294735

ABC294735 inhibits both SphK1 and SphK2 with an IC_50_ ~10 μM [162]. In combination with sorafenib, ABC294735 exerts cytotoxicity in BxPC-3 and A498 cells along with the significant reduction in ERK phosphorylation. The combination of ABC294735 and sorafenib also delays tumorigenesis in xenograft models [66,163].

### 7.7. ABC294640

ABC294640 is a specific and competitive inhibitor of SphK2 (IC_50_ = 9 μM) which significantly prevents its off-target inhibition of protein kinases. ABC294640 reduces intracellular S1P levels with concomitant elevation of ceramide in tumor cells. It also suppresses pERK and Akt signaling and promotes apoptosis and autophagy in tumors [226,227,228,229]. Lewis et al. [164] showed that the administration of ABC294640 downregulates c-Myc and RRM2 expression thereby attenuating tumor proliferation in human pancreatic cancer cell lines (BxPC-3, MiaPaCa-2, and Panc-1), increases their sensitivity towards gemcitabine. The phase I clinical trial of ABC294640 (*NCT01488513*) in patients with cholangiocarcinoma has been completed, demonstrates the pharmacological inhibition of SphK2 subsequently causes caspase-dependent apoptosis and attenuation of tumor growth [165]. Clinical trials of ABC294640 in other tumor types have been started which were either withdrawn or terminated. This list includes patients with refractory/relapsed diffuse large B-cell lymphoma or Kaposi sarcoma (ClinicalTrials.gov; *NCT02229981*), hepatocellular carcinoma (ClinicalTrials.gov; *NCT02939807*) and multiple myeloma (ClinicalTrials.gov; *NCT02757326*) [228].

### 7.8. MP-A08

MP-408 is an ATP-competitive inhibitor, targeting both SphK1 and SphK2 selectively over other kinases. It was discovered using a combined approach of high-throughput screening of small molecule libraries and homology modeling of the ATP-binding pocket of SphK1 followed by molecular docking. MP-408 functions by blocking pro-proliferative signaling cascades and induces apoptosis in an SphK-dependent manner. It represses tumorigenesis in the human lung adenocarcinoma in the mouse xenograft model by inducing mitochondrial-associated cell death and blocking angiogenesis [66,166]. 

### 7.9. Amgen-82

By combining the structures of sphingosine and SKI-II, scientists at Amgen (Thousand Oaks, CA, USA) designed a structural scaffold, the (2*R*,4*S*)-2-(hydroxymethyl) piperidin-4-ol moiety, which was further modified to develop a series of compounds targeting SphKs. Among all the members, the 82nd compound, named Amgen-82, showed inhibitory action towards both the isoforms of SphK, with different selectivity. Amgen-82 elicit good pharmacokinetic feature with enhanced selectivity towards SphK1 (IC_50_ = 70 nM) over SphK2 (IC_50_ > 10 μM) [167]. Amgen 82 inhibits SphK activity and reduces the S1P intracellular levels but does not significantly affect cell viability at IC_50_ concentration in cancer cell lines. Cell death was observed only when Amgen-82 is administered at a higher concentration attributed to its detergent-like physicochemical properties. Moreover, the administration of Amgen-82 did not significantly affect the tumor growth in the xenograft mice model despite decreasing S1P circulatory levels [168,211].

### 7.10. Natural Compounds

Natural compounds isolated from plants and microorganisms possess antioxidant, anti-diabetic, anti-inflammatory, hepatoprotective and anti-cancer properties. Natural products have been used for a long to combat human diseases [230,231,232,233]. Researchers have utilized their structures as a scaffold to design new drugs with improved therapeutic potential and pharmacokinetics profile.

Several natural compounds, especially phytochemicals from plants, have been identified as SphK/S1P signaling cascade inhibitors. For instance, quercetin improves pulmonary fibrosis in mice models by inhibiting SphK1/S1P signaling [48]. Resveratrol inhibits SphK activity and prevents its translocation to the plasma in C5 anaphylatoxin stimulated neutrophils [234]. Chowdhury et al. [235] showed that epigallocatechin-3-gallate inhibits U46619-induced phosphorylation and activation of SphK and abrogates intracellular S1P levels. Recently, we showed the inhibition of kinase activity of SphK1 by quercetin and ellagic acid [68,69]. These phytoconstituents exert antiproliferative effects in adenocarcinoma of human alveolar basal epithelial cells (A549). Icaritin, a flavonoid, was observed to inhibit SphK1, resulting in cytotoxicity and enhanced apoptosis in hepatocellular carcinoma cell lines (Huh-7, HepG2, and KYN-2) that further hinders tumorigenesis [171]. Hispidulin, another flavanoid, was shown to inhibit cell proliferation and invasion in renal carcinoma cell lines (A498 and Caki-2) by inhibiting SphK1 [172].

Funaki et al. [173] reported that a derivative of vitamin A, peretinoin, downregulates the expression of SphK1 in a hepatocellular carcinoma cell line (Huh-7) by decreasing the activity of SphK1 promoter. Luteolin, another plant flavonoid, elicits cytotoxic effects on colon cancer cells by inhibiting SphK2 with the concomitant reduction in intracellular S1P levels [236]. The ethanolic extract of *Amomum tsaoko*, a Chinese spice, is observed to inhibit SphKs. Chemical processing of the extract led to the isolation and identification of various compounds, including phenolics, fatty acids, terpenoids, and aliphatic alcohols. Two compounds exhibited inhibitory action by binding to the substrate-binding pocket of both the isoforms of SphK [237]. Lee et al. [174] showed the anticancer effects of pristimerin, a triterpenoid quinone methide, that inhibits the HIF-1α accumulation via the inactivation SphK1 signaling cascade in a hypoxic prostate cancer cell line (PC-3). Moreover, pristimerin-induced cancer stem cell toxicity is seen in breast cancer stem cells and esophageal squamous cell carcinoma [238]. 

In addition to phytochemicals, some compounds obtained from marine organisms, such as B-5354a, b, and c and pachastrissamine (PA) and its derivatives, also manifest good inhibitory potential towards PhDs [175]. PA displays cytotoxic activity and accelerates apoptosis in A549 and melanoma cells [176]. PA exerts its anticancer effects by inhibiting the phosphorylation of ERK and FOXO3 in melanoma cells. Moreover, PA administration in a C57BL/6 mouse suppresses melanoma cell growth without imparting significant toxic effects [177]. Although, the SphK inhibitors from natural sources are potent with IC_50_ value ranging in the micromolar range, their selectivity, bioavailability and large-scale production are still largely unknown. Hence, an area of active research in the scientific community.

## 8. Antagonists of S1P Receptors

### 8.1. Suramin

Suramin, a derivative of urea, is an antagonist of S1PR3. S1P/S1PR3 signaling promotes the migration of bone marrow cells to damaged liver tissue and thus plays a vital role in liver fibrosis. Li et al. [178] showed the inhibition of bone marrow cell migration and homing by suramin in cholestasis-induced liver fibrosis mice model. Suramin administration leads to a significant reduction in bone marrow-derived cells during cholestasis and decreases the bile duct ligation induced liver fibrogenesis. Lustberg et al. [179] tested the efficacy of suramin efficacy combined with paclitaxel in phase I/II clinical trials of metastatic breast cancer. They demonstrated that the mixture of non-cytotoxic doses of suramin and paclitaxel was well tolerated and elicit anti-tumor activity. In combination with other anti-cancer drugs, the suramin is under clinical trials for many cancer types, including prostate, breast, lung, bladder, and brain carcinomas (ClinicalTrials.gov).

### 8.2. VPC23019

VPC23019 serves as a lead compound in a series of aryl-amide-containing compounds that act as competitive antagonists for S1PR1 and S1PR3. VPC23019 interferes with the activation of these S1PRs by S1P [180]. Wang et al. [239] demonstrated that pretreatment of VPC23019 inhibits the S1P-induced proliferation in endothelial progenitor cells. They observed that VPC23019 promotes the activation of caspa[se-3, leading to enhanced apoptosis in endothelial progenitor cells via the S1PR1/S1PR3/PI3K/AKT pathway. Furthermore, Dai et al. [181] showed that pretreatment of VPC23019 significantly inhibits endothelial cell migration and invasion in ovarian cancer cells (SKOV3 and HO8910PM). The blockade of S1PR1/3 by VPC23019 suppresses tumor growth, angiogenesis, and angiogenic factor expression in ovarian cancer cells. The analogs of VPC23019, developed by shortening alkyl chain, worked as antagonists of S1PR1 and S1PR3. Davis et al. [180] demonstrated that VPC23019 analogs inhibit the agonist-induced calcium mobilization and migration in urinary bladder carcinoma cell lines (T24 cells). 

## 9. S1P-Blocking Antibodies

Sonepcizumab is the first monoclonal antibody that targets explicitly S1P. Sonepcizumab has been shown to inhibit angiogenesis, metastasis and reduces tumor volume in a xenograft mouse model of various tumor types, including lung, ovary, breast, and melanoma [192,193]. Sphingomab, the murine counterpart of sonepcizumab, significantly reduces the tumor-associated migration and angiogenesis in ovarian SKOV3 and lung A549 xenograft models in subcutaneous murine melanoma B16-F10 allograft [194,195]. ASONEP, a formulation of sonepcizumab that serves as a potential drug candidate, has completed phase I clinical trials to treat advanced solid tumors (*NCT00661414*) [192].

## 10. Conclusions and Future Perspectives

Given the critical role of SphK/S1P in cancer progression and various other inflammatory diseases, targeting SphK/S1P signaling pathway is an attractive therapeutic strategy. We report in this recent review findings that have highlighted the possible role of SphK/S1P in tumorigenesis, advocating as a novel drug target for cancer therapy. The review provides an overview of the compounds that target the SphK1/S1P signaling axis and are currently at different stages of clinical trials.

Despite a wealth of literature, some studies contradict the well-established role of SphK and S1P in cancer and their targeting for cancer therapy. For instance, Rex et al. [168] showed that Amgen82, an SphK1 inhibitor, reduces the S1P intracellular levels; it does not affect cell viability and tumor volume therapeutically safer concentrations in cancer cell lines and mice model. Schnute et al. [201] demonstrated no significant effect on tumorigenesis upon targeted inhibition of SphK1 by PF453 in head and neck carcinoma cells. In another study, Kharel et al. [240] reported that the inhibition of SphK1 had reduced S1P levels, cannot always be correlated with changes in cell proliferation and survival. 

It is a well-known fact that cancer is a complex disease involving dysfunction in multiple systems. Even after the SphK/S1P axis blockade, the cancer cells may still survive because of other signaling pathways in the network that can circumvent SphK/S1P inhibition’s harmful consequences. It is essential to keep in mind that pharmacological validation of the distinct roles of SphK, S1P, and S1PRs in cancer progression is highly sought to make further progress in this field. Hence, extensive studies on SphK/S1P signaling axis in correlation with cancer and other human diseases need to be performed to provide indisputable and consolidated evidence for considering SphK/S1P as druggable targets. 

To date, no SphK/S1P inhibitor has been used for cancer treatment. Thus, more elaborative research about the development of high affinity and high specificity SphK inhibitors should be followed up. We anticipate that the crystal structures of SphK2 and other S1PRs would be determined soon to get deeper insights into the structure-function relationships and the designing of potent and selective inhibitors. Structural information will further help understand the SphK/S1P signaling axis in the rational design with an appreciable drug-like profile. Furthermore, comprehensive research focusing on a better understanding of SphK/S1P sphingolipids in various human diseases should be carried out. Undoubtedly, such studies will build an improved platform for developing therapeutics against multiple human disorders linked to sphingolipid metabolism, particularly SphK/S1P signaling cascade.

## Figures and Tables

**Table 1:** Overview of compounds that target SphK/S1P/S1PR axis in cancer.

Class	Compound	Target (IC_50_)	Specificity	Experimental Model	Highest Testing Phase	Effects	References
Inhibitors of SphK1	SKI-I	SphK1 (10 µM)	Yes	Acute myeloid leukemia cells	Preclinical	Induces apoptotic cell death. Decreases cancer progression, angiogenesis, and lymphangiogenesis	[140,141]
	SKI-II	SphK1 (16 µM) SphK2 (8 µM)	No	HepG2 cells BDL or CCl_4_-treated mice model of hepatic fibrosis	Preclinical	Inhibits tumorigenesis in xenograft and fibrosis mice model. Induces apoptosis with antiproliferative effects in different cancer cell lines	[142,143]
	PF-543	SphK1 (3.6 nM)	Yes	Hepatic fibrosis mice model, Colorectal carcinoma, head, and neck squamous carcinoma and breast cancer cells.	Preclinical	Inhibits fibrogenesis in hepatic fibrosis mice model and human hepatic stellate cells. Inhibits cell proliferation and imparts cytotoxicity in various cancer cell lines	[144,145,146,147]
	DMS	SphK1, SphK2 (<1.0 mM)	No	Histiocyticlymphoma, Jurkat and leukemia, lung, breast, hepatocellular carcinoma (SK-Hep1 and MHCCLM3) cells	Preclinical	Induces apoptosis and delays cancer progression in various human cancer cell lines	[148,149,150,151]
	DHS (Safingol)	SphK1 (5 μM)	No	Jurkat and leukemia cells (U937), metastatic solid tumor	Phase I clinical trial in combination with cisplatin for solid tumors	Decreases cell proliferation, invasion and inhibits tumorigenesis	[152,153]
	FTY720 (Fingolimod) S1P receptor-independent	SphK1 (5.0–12.5 μM)	No	Liver cancer, breast cancer, bladder cancer, glioblastoma, leukemia, and malignant mesothelioma cells	FDA-approved and launched for clinical use (2010) to treat multiple sclerosis	Decreases colitis and cancer progression Antiproliferative and antimetastatic effects in various cancer cell lines.	[154,155,156,157,158,159,160]
	RB-005 and RB-019	SphK1 (IC_50_ = 3.6 µM)	Yes	Proliferating human pulmonary arterial smooth muscle cells (PASMC)	Biological testing	Inhibits proliferative diseases, including PAH (pulmonary arterial hypertension)	[161]
	ABC294735	SphK1, SphK2 (10 μM)	No	Pancreatic cancer cell s (BxPC-3) and Renal cancer cells (A498) (combination with Sorafenib)	Preclinical	Delays tumorigenesis in xenograft models. Exerts antiproliferative and cytotoxic activity in cancer cell lines.	[162,163]
	ABC294640	SphK2 (9.0 μM)	Yes	Pancreatic cancer Cholangiocarcinoma cells (HuCCT1, OZ, HuH28, EGI-1, WITT, and LIV27)	Phase I clinical trial for different cancer	Decreases liver transplant graft injury and rheumatoid arthritis. Attenuates tumor proliferation in human cancer cell lines	[164,165]
	MP-A08	SphK1 (6.9 mM) SphK2 (27 mM)	Yes	Human lung adenocarcinoma (A549) xenograft model)	Preclinical	Induces tumor cell apoptosis and inhibits tumor angiogenesis in a mouse xenograft model	[66,166]
	Amgen 82	SphK1 (20 nM) SphK2 (14 nM)	No	Tumor xenograft mice model	Preclinical	Attenuate S1P intracellular levels with no appreciable effect on cell viability at therapeutic concentrations; promotes cell death only at higher concentrations	[167,168]
	EGCG	SphK1 (75 μM)	No	Myeloid leukemia cells (K562, HL-60 and Kasumi-3)	Biological testing	Polyphenon E induces cell death in chronic myeloid acute myeloid leukemia cells. EGCG/safingol combination suppresses viable cell number in Chronic lymphocytic leukemia cell lines	[169,170]
	Icaritin	SphK1	No	Hepatocellular carcinoma cells (Huh-7, HepG2, and KYN-2)	Biological testing	Imparts cytotoxicity, enhances and hinders tumorigenesis in liver cancer cell lines	[171]
	Hispidulin	SphK1	No	Renal carcinoma cells (A498 and Caki-2)	Biological testing	Inhibits cell proliferation and invasion in kidney cancer cell lines	[172]
	Ellagic acid	SphK1	No	Adenocarcinoma alveolar cells (A549)	Biological testing	Imparts cytotoxicity in NSCLC (Non-small cell lung cancer)cell lines	[69]
	Peretinoin	SphK1	No	Hepatoma cell line (Huh-7)	Biological testing	Suppresses cancer progression in liver cancer cell line	[173]
	Pristimerin	SphK1		Prostate cancer cell line (PC-3), breast cancer stem cells and esophageal squamous cell carcinoma (ESCC)	Biological testing	Inhibits HIF-1α accumulation in a hypoxic prostate cancer cell line. Imparts toxicity in cancer stem cells	[174]
	Pachastrissa mine	SphK1, SphK2 (4.6 µM)	No	Skin cancer cells (A375 and B16F10 cell), Melanoma tumor xenograft mice model	Biological testing	Imparts cytotoxicity and accelerates apoptosis in melanoma cell lines. Suppresses melanoma cell growth in mice model	[175,176,177]
Antagonists and Agonists of S1P receptors	Suramin	An antagonist of S1PR3 and S1PR5 (50–100 µM)	Yes	CCl4/BDL-induced liver fibrosis in mice	Phase I/II clinical trial	Impedes liver fibrogenesis Elicits anti-tumor activity in breast cancer cells	[178,179]
	VPC23019 and its analogs	Antagonists of S1PR1 (50 nM) and S1PR3 (100 nM)	Yes	Endothelial progenitor cells, ovarian cancer cells and urinary bladder carcinoma cell line Ovarian cancer xenograft mice model	Preclinical	Inhibits proliferation and promotes apoptosis in endothelial progenitor cells. Inhibit the endothelial cell migration, invasion, tumor growth and angiogenesis in cancer cell lines	[180,181]
	FTY720 (Fingolimod)	S1PR1 agonist and functional antagonist, an Agonist of S1PR3, S1PR4, and S1PR5 (=0.2–6 nM)	Yes	Liver, breast, prostate, bladder, leukemia, colon and ovarian cancer cell lines Xenograft mouse model of hepatocellular carcinoma, bladder and lung cancer	FDA-approved drug to treat multiple sclerosis	Imparts immunosuppressive effects Arrests growth and induces apoptosis in cancer cell lines. Suppresses tumor growth in various mice xenograft models	[155,158,182,183,184,185,186,187,188]
	Ozanimod (RPC1083)	S1PR1 (0.16 nM) and S1PR5 agonist	Yes	LNCaP, PC-3, and DU-145 cellular models of prostate cancer	FDA-approved for the treatment of relapsing forms of multiple sclerosis, under evaluation for use in ulcerative colitis and Crohn’s disease in multinational phase III trials, under pre-clinical evaluation for anticancer effects in cellular models of prostate cancer	Inhibits the colony formation and migratory characteristics of PCa cells. Induces apoptosis in PC-3 and DU-145 cells	[189,190]
	Siponimod (BAF312)	S1PR1 and S1PR5 modulator	Yes		Recommended for secondary progressive multiple sclerosis		[191]
S1P-blocking antibodies	ASONEP^TM^ (sonepcizumab/LT1009)	S1P-blocking antibody (IC_50_ = 100 pM)	Yes	Subcutaneous murine melanoma B16-F10 allograft, human lung A549 and ovarian SKOV3 xenograft models	Phase I clinical trial for solid tumors	Reduces the tumor-associated migration and angiogenesis in murine xenograft and allograft model	[192,193,194,195]

## Data Availability

Not Applicable.
